# Health Outcomes Associated with Adherence to Antidepressant Use during Acute and Continuation Phases of Depression Treatment among Older Adults with Dementia and Major Depressive Disorder

**DOI:** 10.3390/jcm9103358

**Published:** 2020-10-20

**Authors:** Sandipan Bhattacharjee, Suniya Naeem, Shannon M. Knapp, Jeannie K. Lee, Asad E. Patanwala, Nina Vadiei, Daniel C. Malone, Wei-Hsuan Lo-Ciganic, William J Burke

**Affiliations:** 1 Health Outcomes Division, College of Pharmacy, University of Texas at Austin, Austin, TX 78712, USA; 2 Department of Psychiatry, University of Kentucky, Bowling Green, KY 42101, USA; snaeem94@gmail.com; 3 Statistics Consulting Laboratory, Bio5 Institute, The University of Arizona, Tucson, AZ 85721, USA; sknapp@statlab.bio5.org; 4 Department of Pharmacy Practice and Science, College of Pharmacy, The University of Arizona, Tucson, AZ 85721, USA; jlee@pharmacy.arizona.edu (J.K.L.); vadiei@pharmacy.arizona.edu (N.V.); 5 Faculty of Medicine and Health, School of Pharmacy, Royal Prince Alfred Hospital, S343, Pharmacy Building (A15), The University of Sydney, Sydney NSW 2006, Australia; asad.patanwala@sydney.edu.au; 6 Department of Pharmacotherapy, Skaggs College of Pharmacy, University of Utah, Salt Lake City, UT 84112, USA; Dan.Malone@utah.edu; 7 Department of Pharmaceutical Outcomes & Policy, College of Pharmacy, University of Florida, Gainesville, FL 32610, USA; wlociganic@cop.ufl.edu; 8 Banner Alzheimer’s Institute, Phoenix, AZ 85006, USA; wjburkemd@gmail.com

**Keywords:** dementia, depression, antidepressants, adherence, all-cause mortality, all-cause hospitalization, falls/fractures

## Abstract

Objectives: To examine health outcomes associated with adherence to Healthcare Effectiveness Data and Information Set (HEDIS) antidepressant medication management (AMM) during acute and continuation phases of depression treatment among older adults with dementia and major depressive disorder (MDD). Design: Retrospective cohort study. Setting: Medicare 5% sample data (2011–2013). Participants: Older adults (aged 65 years or older) with dementia and MDD. Measurements: The first antidepressant prescription claim from 1 May 2011 through 30 April 2012 was considered the index prescription start date (IPSD). Adherence during acute- and continuation-phase AMM was based on HEDIS guidelines. Study outcomes included all-cause mortality, all-cause hospitalization, and falls/factures (with mortality being the competing event for hospitalization and falls/fractures) during follow-up from end of acute-/continuation-phase AMM adherence. Due to the proportionality assumption violation of Cox models, fully non-parametric approaches (Kaplan–Meier and modified Gray’s test) were used for time-to-event analysis adjusting for the inverse probability of treatment weights. Results: Final study samples consisted of 4330 (adherent (N) = 3114 (71.92%)) and 3941 (adherent (N) = 2407 (61.08%)) older adults with dementia and MDD during acute- and continuation-phase treatments, respectively. No significant difference (*p* > 0.05) between adherent and non-adherent groups was observed for all-cause mortality and falls/fractures in both the acute and continuation phases. There was a significant difference in time to all-cause hospitalization during acute-phase treatment (*p* = 0.018), with median times of 530 (95% CI: 499–587) and 425 (95% CI: 364–492) days for adherent and non-adherent groups, respectively. Conclusions: Acute-phase adherence to HEDIS AMM was associated with reductions in all-cause hospitalization risk among older adults with dementia and MDD.

## 1. Introduction

Dementia is a public health priority with a substantial economic burden (costs amounted to $290 billion in 2019) and is associated with several neuropsychiatric comorbidities, depression being the most common [1,2]. Evidence suggests that the coexistence of dementia and depression causes greater functional impairment than either condition alone, having negative impact on patients and families, and burdening the healthcare system further due to the greater need for these patients to be placed in nursing homes [3].

While treatment guidelines for depression in adults are well established, studies exploring the outcomes of antidepressant use among older adults with dementia have yielded conflicting findings. A number of studies have shown that antidepressant use in patients with dementia led to improvements in functional status [4,5,6]. Additionally, a recent 2019 study conducted in the Taiwanese population found that most antidepressant treatments had significantly protective effects on all-cause mortality [7]. Conversely, other studies have reported little to no statistically significant correlations between antidepressant use in dementia and favorable outcomes [8,9]. A meta-analysis published in 2018 investigated the efficacy and safety of antidepressants for patients diagnosed with dementia and showed little to no improvements in depression rating scale scores [10]. These mixed findings emphasize the need to conduct a comprehensive study exploring the effects of antidepressant treatment on various healthcare outcomes among older adults with concurrent dementia and depression.

In the present study, we examined the association of real-world outcomes (all-cause mortality, all-cause hospitalization, and falls/fractures) with adherence to antidepressant therapy during acute and continuation phases of depression treatment using a nationally representative sample of United States (U.S.) older adults with dementia and newly diagnosed major depressive disorder (MDD). Currently, Healthcare Effectiveness Data and Information Set (HEDIS) recommendations for antidepressant medication management (AMM) are used to guide treatment for depression across all populations [11]. However, these guidelines are not tailored to account for potentially inappropriate antidepressants for older adults; hence, we excluded antidepressants that were deemed to be potentially inappropriate based on the Beers [12] and Screening Tool of Older Persons’ potentially inappropriate Prescriptions (STOPP) [13] criteria in this study. Our previous study [14] provides details regarding the potentially inappropriate antidepressant use in older adults.

## 2. Materials and Methods

### 2.1. Study Design

**Figure 1:**
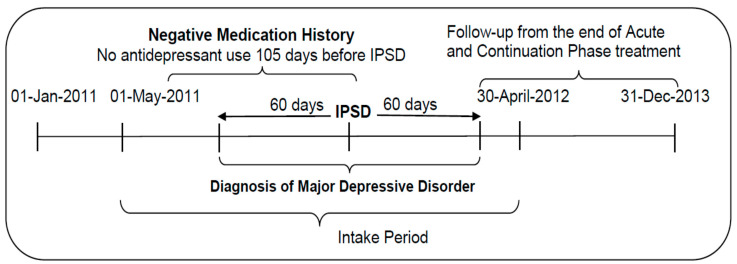
Diagram of the study design.

We conducted a retrospective, cohort study. Figure 1 depicts the design of this study.

### 2.2. Data Sources

The primary data source used in this study was the Medicare 5% sample claims data from 2011 to 2013, including claims from the inpatient, outpatient, skilled nursing facility, carrier, hospice care, home health agency, durable medical equipment, and Part D event (PDE) Standard Analytic Files. Demographic information (e.g., age, gender, race/ethnicity) as well as information on eligibility, residence (state and county), and date of death, were obtained from the Medicare Beneficiary Summary File (MBSF). A 5% random sample of the Medicare beneficiaries in the U.S. is included in the Medicare 5% sample claims data making it a nationally representative sample of the Medicare beneficiaries in the U.S. The current study utilized de-identified Medicare data that were obtained from the Centers for Medicare and Medicaid Services (CMS). A strict data use agreement (DUA) was set up between CMS and The University of Arizona to properly use the Medicare data. According to the CMS policy, the Medicare data we used for the current study cannot be shared with other individuals/groups/organizations without proper DUA approval. Hence, we will not be able to publicly share the Medicare claims data with any individuals/groups/organizations without CMS approval. However, interested parties can feel free to contact the first/corresponding author of this study (S.B.) via email to obtain information regarding the DUA process for acquisition of the Medicare data.

Characteristics of the subjects’ county of residence (including median income and number of medical providers) were obtained from the Area Health Resource File, a publicly available, county-specific database. The specialty of healthcare providers was obtained from the National Plan and Provider Enumeration System (NPPES); this file contains the National Provider Identifier (NPI), a unique identification number issued to healthcare providers by CMS, which we used to link with the NPIs in the PDE file.

### 2.3. Study Sample

The study sample consisted of older adults aged 65 years and older with a diagnosis of dementia based on the Chronic Condition Data Warehouse (CCW) algorithm [15]. Based on the HEDIS guidelines [11], the intake period for new antidepressant medication use was from 1 May 2011, through 30 April 2012; the index prescription start date (IPSD) was the first date of an antidepressant prescription claim during the intake period. The 105 days preceding the IPSD was considered “baseline”. Index antidepressants included in this study excluded those deemed potentially inappropriate for older adults by the Beers [12] and STOPP [13] criteria. Medicare beneficiaries with a pharmacy claim for either new or refill prescriptions for an antidepressant medication during the baseline were excluded from the study sample (negative medication history). Additionally, only older adults with dementia and a concurrent diagnosis of MDD in claims during the 121 day period, from 60 days before the IPSD through the IPSD and 60 days after the IPSD, were included in the final study sample.

MDD was ascertained by HEDIS recommendations of using primary or secondary International Classification of Diseases, Ninth Revision, Clinical Modification (ICD-9-CM) codes (296.2, 296.3, 309.1, 300.4, and 311) from inpatient/outpatient claims [11]. Several prior studies have used this definition to identify MDD [16,17,18,19]. Inclusion also required having continuous Medicare enrollment during baseline and during the 114 days post-IPSD (for the acute-phase adherence group) or during the 231 days post-IPSD (for the continuous-phase adherence group). Medicare beneficiaries were excluded from the final study sample if they (i) died during the 114 days post-IPSD (for acute phase adherence group) or during the 231 days post-IPSD (for continuous phase adherence group); (ii) were enrolled in Health Maintenance Organizations during baseline or during the respective post-IPSD acute- and continuous-phase treatment; (iii) had a fall or fracture during baseline; (iv) had a prescription for an inappropriate antidepressant during the 114 days post-IPSD (for the acute-phase adherence group) or during the 231 days post-IPSD (for the continuous-phase adherence group); (v) had end-stage renal disease (ESRD) any time during the calendar year of IPSD; (vi) were diagnosed with end-stage liver disease (ESLD) during baseline; or (vii) had missing race/ethnicity information. ESRD was identified from the Medicare Beneficiary Summary File (MBSF), whereas ESLD was identified using ICD-9-CM codes of 155.0 and 571.0-9 [20]. Subjects included in the continuation-phase adherence analysis represent a subset of those included in the acute-phase adherence analysis.

### 2.4. Key Independent Variables

The key independent variables were adherence during acute-phase treatment and adherence during continuation-phase treatment. Based on HEDIS guidelines [11], adherence during acute-phase treatment required prescription coverage for at least 85 days of the first 115 of treatment (starting with the IPSD); those failing to meet this threshold were classified as “non-adherent” for the acute period. Similarly, adherence during continuation-phase treatment required prescription coverage for at least 181 days of the first 232 of treatment (starting with the IPSD); those failing to meet this threshold were classified as “non-adherent” for the continuation period.

Subjects who were considered “adherent” for the acute period may not have been adherent during the continuation period. Whether or not a subject was covered by their antidepressant prescription for a given day was calculated based on the date and days of supply of antidepressants from prescription claims in the PDE file (details of the algorithm used are available elsewhere [21]).

### 2.5. Outcomes of Interest

Outcomes of interest included time to all-cause mortality, time to first all-cause hospitalization, and time to first fall or fracture, with time measured from the end of the respective adherence period (acute or continuation). For hospitalization and fall/fracture outcomes, death was treated as a competing event. Mortality was identified from the MBSF files. Hospitalization was identified from the inpatient claims data. Falls and fractures were identified using validated ICD-9-CM and current procedural terminology codes based on the Tseng et al. study [22].

### 2.6. Inverse Probability of Treatment Weighting (IPTW)

To account for baseline differences in characteristics between adherent and non-adherent groups that might affect the outcomes, we used IPTW for all analyses. Propensity scores for each individual were calculated via logistic regression with the following predictor variables: gender (female or male); age (65–74 or ≥75 years old); race (White or non-White); receipt of public assistance (indicated by Medicare premiums and deductibles that were subsidized for the enrollee by the state); census region (Northeast, South, Midwest, or West); metropolitan residency status (yes or no); whether or not there was a diagnosis of Parkinson’s disease during baseline (indicated by the presence of ICD-9-CM code 332.x); psychotherapy during baseline (yes or no); the specialty of the provider associated with the index prescription (general family, psychiatry, neurology, other, or unknown); the per-capita density by county of each of neurologists and psychologists (classified into 4 groups: 0 and tertiles for values >0); Elixhauser Index based on diagnoses present during baseline (truncated at 3); and the presence or absence of prescriptions for each of the following medication types during baseline: angiotensin-converting-enzyme (ACE) inhibitors, angiotensin II receptor blockers (ARBs), anticoagulants, antidiabetics, anti-Parkinsonian, antipsychotics, anxiolytics, beta-blockers (BBs), calcium-channel blockers (CCBs), diuretics, proton pump inhibitors (PPIs), and statins.

As the analyses for acute-phase adherence and for continuation-phase adherence involved different sets of subjects, propensity scores (and the respective IPT weights) were calculated separately for each subset. We calculated standardized mean differences (SMDs) for each of the variables used in propensity score calculation before and after IPTW adjustment and considered an SMD of <0.20 [23] as indicative of achieving good balance after IPTW adjustment.

### 2.7. Statistical Analysis

Due to violation of the proportionality assumption inherent in Cox models, we opted to employ fully non-parametric analyses. For all-cause mortality, survival was estimated using the Kaplan–Meier model and the weighted log-rank test was used to test of differences in survival curves between adherent and non-adherent groups. For hospitalization and falls/fractures outcomes, cumulative incidence functions (CIF) were plotted, and the modified Grey’s test [21] was used to compare the curves between adherent and non-adherent groups. For all outcomes, observations were censored at the time of discontinuation of continuous Medicare coverage or addition of Health Maintenance Organizations (HMO) coverage if these occurred prior to the outcome of interest.

Point estimates and 95% CIs for survival and cumulative incidence of hospitalization and falls/fractures were calculated at 90, 180, 270, 365, 455, and 545 days. We also calculated point estimate and 95% CIs of the first quartile and median (where possible) of time to each outcome. For hospitalization and falls/fractures data, confidence intervals were calculated via bootstrap sampling. Analyses for all-cause mortality were conducted in SAS (SAS 9.4, PROC LIFETEST, SAS Institute, Cary, NC, USA) and analyses for hospitalization and falls/fractures were conducted in R (v. 3.5.1, R Foundation for Statistical Computing, Vienna, Austria) [24]. Statistical significance was set at α = 0.05.

### 2.8. Sensitivity Analysis

During sensitivity analysis, we excluded subjects with physician specialty unknown; propensity scores and the respective IPTWs were recalculated for these subsets (acute- and continuation-phase treatment groups).

## 3. Results

**Figure 2:**
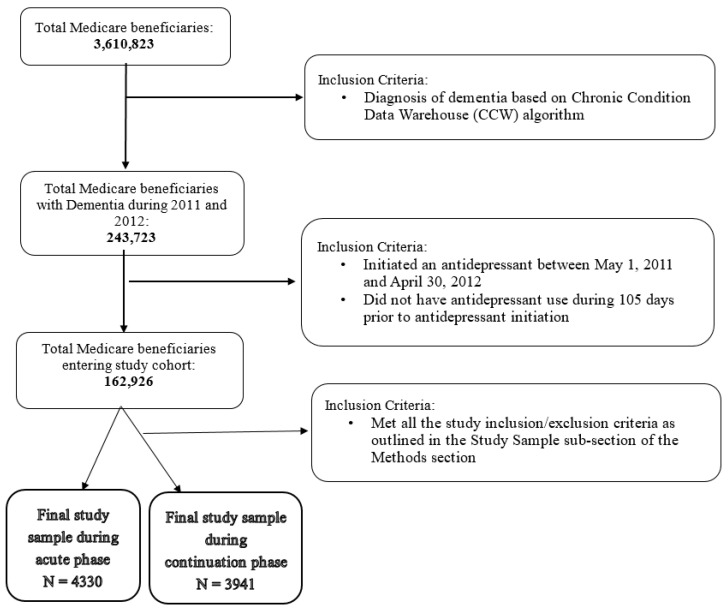
Cohort development for base case analysis.

Figure 2 shows the development of our final study sample. After applying all study inclusion/exclusion criteria, our final study samples consisted of 4330 (adherent (N) = 3114 (71.92%)) and 3941 (adherent (N) = 2407 (61.08%)) older adults with concurrent dementia and MDD during acute and continuation phase treatments, respectively.

Table 1 and Table 2 present the baseline characteristics and their differences before IPT-weighting and the *p*-values after IPT-weighting between adherent and non-adherent groups in the acute- and continuation-phase depression treatment groups, respectively. For the study sample included in the acute-phase depression treatment cohort, significant differences for baseline characteristics were observed for race/ethnicity, density of neurologists, Elixhauser comorbidity score, and antipsychotic use. For example, White older adults with dementia and newly diagnosed MDD were more adherent compared to the non-White race/ethnic group (72.90% vs. 65.71%, chi-square test: χ^2^ = 13.083, df = 1, *p*-value < 0.001) (Table 1). However, after IPT-weighting, there were no significant differences in any of the baseline characteristics. Similarly, for the continuation-phase depression treatment cohort, baseline characteristics that were significantly different between adherent and non-adherent groups were race/ethnicity, geographical region, provider specialty, Elixhauser comorbidity score, antipsychotic and anxiolytic use. For example, baseline antipsychotic users were more adherent to treatment compared to non-users (67.49% vs. 59.72%, chi-square test: χ^2^ = 14.386, df = 1, *p*-value < 0.001) (Table 2). After applying IPT-weighting, none of the baseline characteristics were significantly different between the adherent and non-adherent groups. All of the baseline characteristics post IPT-weighting were considered balanced as evidenced by an SMD of <0.20 after IPT-weighting (data not provided in tabular form). Propensity score distribution for adherent and non-adherent groups are presented in Figure S1 (acute) and Figure S3 (continuation), while Figure S2 (acute) and Figure S4 (continuation) depict the distribution of the IPTW.

**Figure 3:**
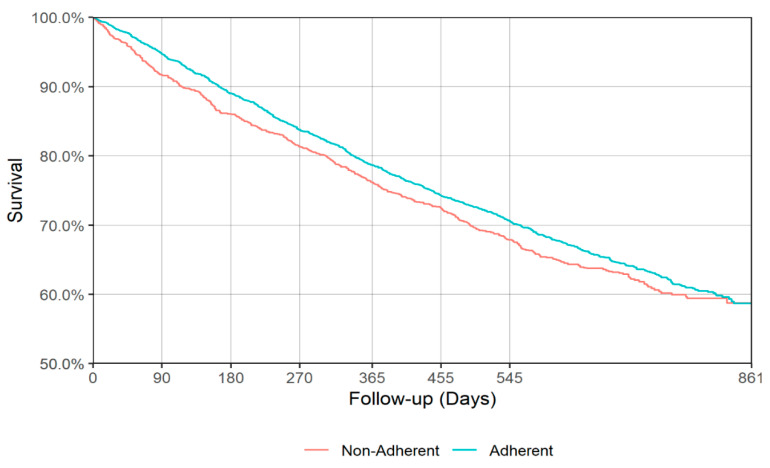
Inverse probability of treatment (IPT)-weighted Kaplan–Meier curve for all-cause mortality (acute phase). Weighted log-rank *p* = 0.168.

**Figure 4:**
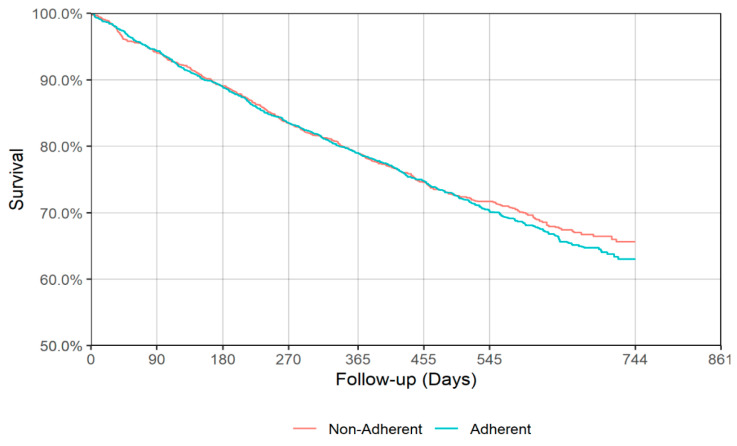
IPT-weighted Kaplan–Meier curve for all-cause mortality (continuation phase). Weighted log-rank *p* = 0.518.

Figure 3 and Figure 4 show the IPT-weighted Kaplan–Meier Curve for all-cause mortality during follow-up from the end of acute and continuation antidepressant treatment phases. The weighted log-rank test did not reveal a statistically significant difference in survival between the adherent and non-adherent group during follow-up for either the end of acute (*p* = 0.168, Figure 3) or continuation (*p* = 0.518, Figure 4) phase of depression treatment. Survival estimates at 90, 180, 270, 365, 455, and 545 days of follow-up from the end of acute or continuation phase of antidepressant treatment for adherent and non-adherent groups are presented in Table 3. The point estimate for the first quartile during the acute phase was 441 (95% CI: 406–474) and 385 (95% CI: 342–455) days for adherent and non-adherent groups, respectively, while for the continuation phase the first quartile time was 445 (95% CI: 415–483) and 443 (95% CI: 397–492) days for adherent and non-adherent groups, respectively (see Table 4).

**Figure 5:**
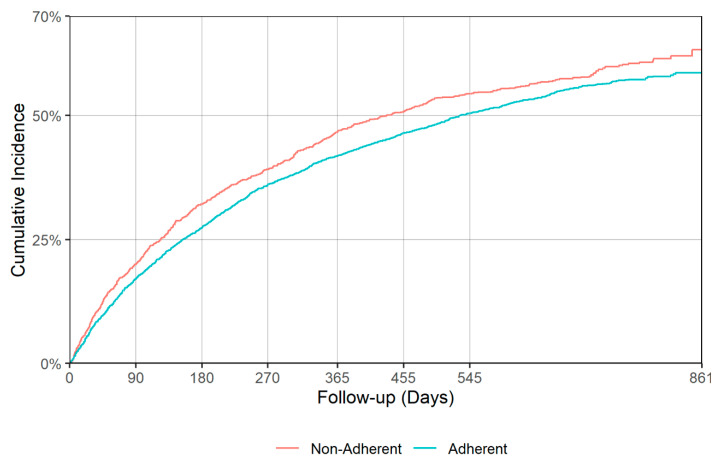
IPT-weighted cumulative incidence function of all-cause hospitalization, adjusting for death before hospitalization as a competing risk (acute phase). Modified Gray’s test *p* = 0.018.

**Figure 6:**
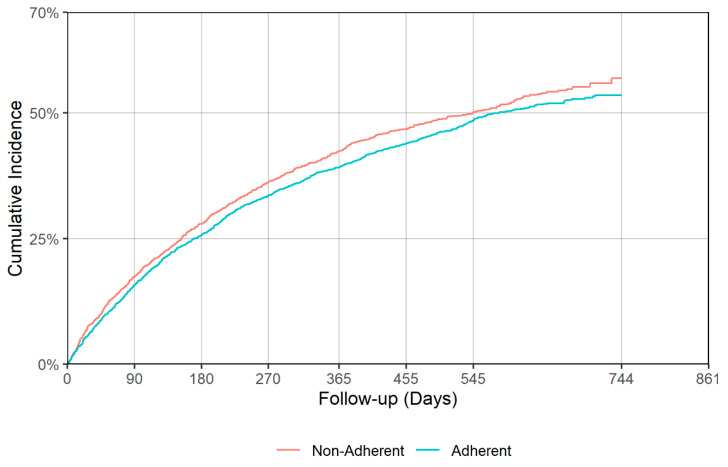
IPT-weighted cumulative incidence function of all-cause hospitalization, adjusting for death before hospitalization as a competing risk (continuation phase). Modified Gray’s test *p* = 0.126.

**Figure 7:**
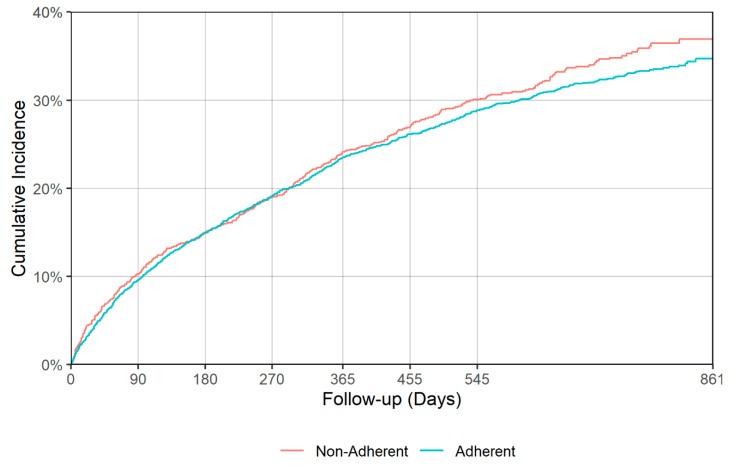
IPT-weighted cumulative incidence function of falls/fractures, adjusting for death before hospitalization as a competing risk (acute phase). Modified Gray’s test *p* = 0.318.

**Figure 8:**
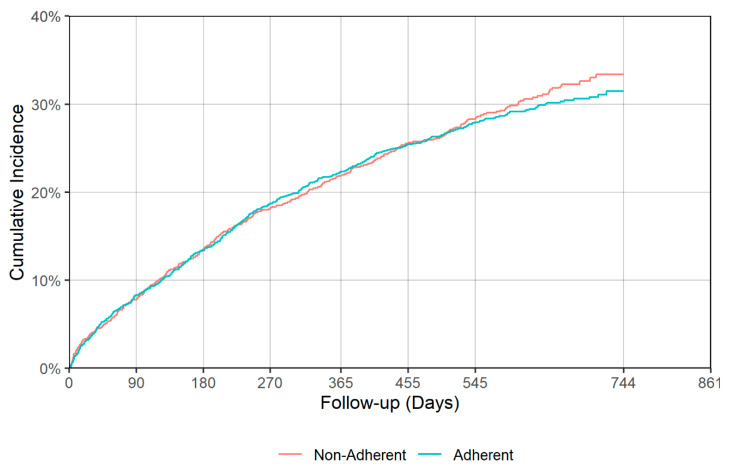
IPT-weighted cumulative incidence function of falls/fractures, adjusting for death before hospitalization as a competing risk (continuation phase). Modified Gray’s test *p* = 0.747.

There was a significant difference in time to all cause hospitalization (*p* = 0.018), with median times of 530 (95% CI: 499–587) and 425 (95% CI: 364–492) days for the adherent and non-adherent groups during acute phase, respectively (Table 4, Figure 5). Delaying all-cause hospitalization is a good indication of antidepressant effectiveness, though no significant difference between adherent and non-adherent groups was observed in terms of all-cause mortality and falls/fractures in either acute or continuation phases. Figure 5, Figure 6, Figure 7 and Figure 8 show the IPT-weighted CIF of all-cause hospitalization and falls/fractures, adjusting for death before hospitalization as a competing risk. CIFs and their 95% CI at 90, 180, 270, 365, 455, and 545 days for hospitalization and falls and fractures, Median and first quartile survival times (95% CI) in days for time-to-hospitalization and falls and fractures (95% CI) are presented in Table 3 and Table 4, respectively.

Sensitivity analyses were conducted by excluding the unknown physician groups since this may have an effect on antidepressant prescribing patterns (as well as adherence), and we observed consistent findings to our base case analysis (details not provided in Tables but available upon request).

## 4. Discussion

Using a U.S. nationally representative study sample, we demonstrated an association between adherence to antidepressants by older adults with dementia and significantly prolonged time to all-cause hospitalization during the acute phase, however, there was no statistically significant association with all-cause mortality or falls/fractures. Findings from this study address the existing knowledge gap regarding the effectiveness of antidepressant treatment among older adults with dementia and MDD by employing a rigorous study design.

The eventual need for hospitalization in older adults with co-existing dementia and depression has been reported in previous studies [25,26,27,28,29]. A National Health and Aging Trends Study conducted with 7179 American older adults (aged 65 and above) found that being hospitalized in the prior year was associated with an odds ratio of 1.42 of probable dementia and an odds ratio of 1.6 of substantial depressive symptoms [25]. However, this study utilized a cross-sectional design, limiting its ability to establish causality. Another study using a nationally representative older population in Taiwan observed that participants with depressive symptoms, cognitive impairment, falls, and urinary incontinence had significantly more hospital admissions (incidence rate ratio = 1.34) and more hospital bed days (incidence rate ratio = 1.72) compared to participants without these conditions [29]. The authors also found the prevalence of aforementioned geriatric syndromes amongst their study population to be 56.3%, highlighting the high healthcare burden. A prospective cohort study using American participants enrolled in the Health and Retirement Study established that depression (Hazard Ratio (HR) = 1.33) and dementia (HR = 1.32) are independently associated with a risk of potentially preventable hospitalizations, and the risk is magnified in adults with comorbid depression and dementia (HR = 1.66) [26]. Our study further stratified these findings between acute- and continuation-phase AMM parameters and found that the only statistically significant difference existed in the acute phase of treatment, with a median time of 530 days in the adherent population and 425 days in the non-adherent population. No significant difference was found for all-cause hospitalization in the continuation phase. This may suggest that early adherence is integral in achieving favorable outcomes and preventing potential hospitalizations, and there may be a specific window of time during treatment in which AMM effects are optimized.

Another variable explored in our study was the effect of antidepressant adherence on all-cause mortality. A cohort study using a nationwide population sample of individuals (age ≥ 60 years) with dementia and depression in Taiwan comparing 18,226 antidepressant users and 7664 non-users of antidepressant found that most antidepressant treatments showed significant protective effects on all-cause mortality, particularly when dosages were optimized (HR = 0.65, 95% CI: 0.62–0.68, *p* < 0.0001) [7]. A longitudinal study investigating the quality of psychopharmacological medication prescribing and its effect on mortality in Medicare beneficiaries in nursing home care showed that the use of appropriate class and duration of antidepressants was associated with a significantly lower mortality risk (HR = 0.8) [5]. Conversely, we were unable to establish a statistically significant relationship between all-cause mortality and antidepressant adherence either in the acute- or in continuation-phase of treatment in our study. Compared with the Su et al. (2019) study [7], we used a more robust study design and employed IPTW to minimize the effects of bias and/or confounding. Moreover, our study used the HEDIS guidelines to define adherence, whereas the Su et al. (2019) study [7], operationalized prescription dosages of antidepressants by cumulative defined daily dosage. Our study results more closely mirror findings from a 2016 study [30] that observed that baseline antidepressant treatment among very old people was not independently associated with increased mortality risk, except when they stratified the data by gender (significant interaction between sex and antidepressant use (HR: 1.76; 95% CI, 1.05–2.94) was observed). This may suggest that gender plays a role in response to antidepressant treatment, and further studies exploring this phenomenon are necessary. Another study [31] conducted among 20,500 individuals diagnosed with incident dementia using the Swedish Dementia Registry (SveDem) showed that being on an antidepressant for >3 years before the diagnosis of dementia significantly decreased the mortality risk, suggesting that the temporality of treatment may additionally be of importance when assessing mortality. Nevertheless, our study did not find a correlation between antidepressant adherence during longitudinal continuation phase and all-cause mortality.

We also assessed the relationship between acute- and continuous-phase AMM and the risk of falls/fractures in our study, which did not show significant associations. While previous studies have independently established causality between falls and medications for depression [32,33,34,35,36,37], there has been a scarcity of studies conducted in patients with concurrent depression and dementia. A 2017 Canadian study among older adults in long-term care reported that the initial stage of treatment with antidepressants led to an increased risk in falls and fall-related injuries [33]. A similar result was described in a study conducted using the Norwegian Prescription Database and Norwegian Hip Fracture Registry [34]. Such risk may be amplified in patients with concurrent dementia due to additional impairments in gait and balance [38]. However, another study that used German primary care patients with dementia showed that using antidepressants for less than six months had no significant impact on the risk of fractures [37]. Few studies that have investigated risk of falls/fractures with antidepressant use among older adults in general, as well as our study sample of co-occurring dementia and depression, interestingly, did not find an increased risk; however, there are many other factors that should be controlled to effectively analyze this association (e.g., severity of dementia, type of setting of these individuals as well as the types of antidepressants being used).

Our study has a number of strengths, including our use of a large nationally representative sample of older Medicare beneficiaries with dementia and depression and the use of the Index Prescription Start Date (IPSD) and Medicare prescription drug information, which allowed us to accurately estimate adherence to medications without being subjected to recall bias. We also utilized a robust study design and multilevel modeling statistical analysis to establish relationships amongst diverse variables. However, some limitations of our study include lack of dementia and depression severity measures within claims data, lack of generalizability to populations outside the U.S., and possible coding errors in the dataset. Due to data limitations, we do not have depression-specific outcomes such as Cornell Scale for Depression in Dementia in this study.

## 5. Conclusions

Acute-phase adherence to HEDIS AMM was associated with significantly prolonging the time to all-cause hospitalization among older adults with dementia and MDD compared to non-adherence; however, no other differences were observed with the other study outcomes between adherent and non-adherent groups. Future large-scale studies are needed to determine optimal adherence thresholds and identify whether this is associated with better outcomes.

## Figures and Tables

**Table 1:** Baseline characteristics of study sample included in the acute depression treatment phase.

Characteristics	Adherent		Non-Adherent		Unweighted	Weighted		Unweighted	Weighted
	N	%	N	%	χ^2^	χ^2^	df	*p*-Value	*p*-Value
Age group					0.283	0.019	1	0.595	0.891
65–74 years	537	71.13	218	28.87					
75+ years	2577	72.08	998	27.92					
Gender					2.592	0.067	1	0.107	0.796
Male	821	70.11	350	29.89					
Female	2293	72.59	866	27.41					
Race/Ethnicity					13.083	0.014	1	<0.001 *	0.906
White	2725	72.90	1013	27.10					
Others	389	65.71	203	34.29					
Public Assistance					0.096	0.004	1	0.757	0.948
Yes	1152	71.64	456	28.36					
No	1962	72.08	760	27.92					
Region					4.275	0.015	3	0.233	1.000
Northeast	629	71.80	247	28.20					
South	1275	71.83	500	28.17					
Midwest	835	73.63	299	26.37					
West	375	68.81	170	31.19					
Metropolitan status					0.132	0.010	1	0.716	0.922
Yes	2413	72.05	936	27.95					
No	701	71.46	280	28.54					
Baseline PD					0.267	0.046	1	0.605	0.831
No	179	73.36	65	26.64					
Yes	2935	71.83	1151	28.17					
Provider Specialty					6.790	0.151	4	0.147	0.997
General/Family	2284	72.60	862	27.40					
Neurology	64	67.37	31	32.63					
Psychiatry	163	73.09	60	26.91					
Unknown	234	72.90	87	27.10					
Other	369	67.71	176	32.29					
Density of Neurologists					9.269	0.023	3	0.026 *	0.999
0	634	71.00	259	29.00					
1	844	73.58	303	26.42					
2	793	68.96	357	31.04					
3	843	73.95	297	26.05					
Density of Psychiatrists					0.324	0.037	3	0.955	0.998
0	507	71.81	199	28.19					
1	866	71.81	340	28.19					
2	850	71.49	339	28.51					
3	891	72.50	338	27.50					
ELX Index					9.101	0.052	3	0.028 *	0.997
0	565	71.25	228	28.75					
1	512	69.10	229	30.90					
2	448	69.46	197	30.54					
3	1589	73.87	562	26.13					
Baseline medication use									
ACE Inhibitor					0.317	0.006	1	0.573	0.938
Yes	862	71.30	347	28.70					
No	2252	72.16	869	27.84					
Anticoagulant					0.481	0.007	1	0.488	0.935
Yes	383	70.66	159	29.34					
No	2731	72.10	1057	27.90					
Antidiabetic					0.728	0.004	1	0.394	0.950
Yes	622	70.76	257	29.24					
No	2492	72.21	959	27.79					
Antiparkinsonian					0.721	0.013	1	0.396	0.908
Yes	209	74.11	73	25.89					
No	2905	71.76	1143	28.24					
Antipsychotic					6.969	0.003	1	0.008 *	0.955
Yes	577	75.82	184	24.18					
No	2537	71.08	1032	28.92					
ARB					1.446	0.000	1	0.229	0.998
Yes	395	74.11	138	25.89					
No	2719	71.61	1078	28.39					
Anxiolytic					5.783	0.049	1	0.016 *	0.825
Yes	395	67.75	188	32.25					
No	2719	72.56	1028	27.44					
Betablocker					0.290	0.007	1	0.590	0.934
Yes	1275	72.36	487	27.64					
No	1839	71.61	729	28.39					
CCB					0.100	0.041	1	0.752	0.840
Yes	754	71.54	300	28.46					
No	2360	72.04	916	27.96					
PPI					0.141	0.001	1	0.708	0.982
Yes	940	72.31	360	27.69					
No	2174	71.75	856	28.25					
Diuretic					1.531	0.000	1	0.216	0.989
Yes	1164	73.02	430	26.98					
No	1950	71.27	786	28.73					
Statin					2.647	0.007	1	0.104	0.934
Yes	1197	73.35	435	26.65					
No	1917	71.05	781	28.95					

Note: Based on 4330 (adherent (N) = 3114 (71.92%)) older adults with dementia and newly diagnosed major depressive disorder (MDD). Abbreviations: PD: Parkinson’s disease; ELX: Elixhauser; ACE inhibitors: angiotensin-converting-enzyme (ACE) inhibitors; ARBs: angiotensin II receptor blockers; CCB: calcium-channel blockers; PPI: proton pump inhibitor. * Represents statistical significance (*p*-value < 0.05).

**Table 2:** Baseline characteristics of study sample included in the continuation depression treatment phase.

Characteristics	Adherent		Non-Adherent		Unweighted	Weighted		Unweighted	Weighted
	N	%	N	%	χ^2^	χ^2^	df	*p*-Value	*p*-Value
Age group					2.298	0.001	1	0.130	0.9715
65–74 years	411	58.55	291	41.45					
75+ years	1996	61.62	1243	38.38					
Gender					2.855	0.012	1	0.091	0.9146
Male	613	58.89	428	41.11					
Female	1794	61.86	1106	38.14					
Race/Ethnicity					38.713	0.000	1	<0.001 *	0.9841
White	2141	63.01	1257	36.99					
Others	266	48.99	277	51.01					
Public Assistance					0.416	0.000	1	0.519	0.9867
Yes	1523	61.46	955	38.54					
No	884	60.42	579	39.58					
Region					15.483	0.007	3	0.001 *	0.9999
Northeast	507	63.45	292	36.55					
South	940	58.86	657	41.14					
Midwest	674	64.81	366	35.19					
West	286	56.63	219	43.37					
Metropolitan status					0.579	0.001	1	0.447	0.9716
Yes	1850	60.76	1195	39.24					
No	557	62.17	339	37.83					
Baseline PD					1.254	0.000	1	0.263	0.9965
Yes	146	64.60	80	35.40					
No	2261	60.86	1454	39.14					
Provider Specialty					19.464	0.059	4	0.001 *	0.9996
General/Family	1766	61.92	1086	38.08					
Neurology	37	44.05	47	55.95					
Other	284	55.91	224	44.09					
Psychiatry	132	62.86	78	37.14					
Unknown	188	65.51	99	34.49					
Density of Neurologists					4.547	0.038	3	0.208	0.9981
0	494	60.76	319	39.24					
1	638	60.99	408	39.01					
2	617	58.99	429	41.01					
3	658	63.51	378	36.49					
Density of Psychiatrists					7.664	0.008	3	0.054	0.9998
0	406	63.84	230	36.16					
1	654	59.19	451	40.81					
2	641	59.13	443	40.87					
3	706	63.26	410	36.74					
ELX Index					10.949	0.017	3	0.012 *	0.9994
0	441	59.12	305	40.88					
1	393	56.79	299	43.21					
2	363	61.01	232	38.99					
3	1210	63.42	698	36.58					
Baseline medication use									
ACE Inhibitor					0.232	0.012	1	0.630	0.914
Yes	676	61.68	420	38.32					
No	1731	60.84	1114	39.16					
Anticoagulant					0.008	0.002	1	0.930	0.9650
Yes	299	60.90	192	39.10					
No	2108	61.10	1342	38.90					
Antidiabetic					3.372	0.000	1	0.066	0.9853
Yes	466	58.25	334	41.75					
No	1941	61.80	1200	38.20					
Antiparkinsonian					0.838	0.000	1	0.360	0.9879
Yes	167	63.74	95	36.26					
No	2240	60.89	1439	39.11					
Antipsychotic					14.386	0.004	1	<0.001 *	0.9478
Yes	463	67.49	223	32.51					
No	1944	59.72	1311	40.28					
ARB					0.167	0.008	1	0.683	0.9276
Yes	300	60.24	198	39.76					
No	2107	61.20	1336	38.80					
Anxiolytic					6.626	0.036	1	0.010 *	0.849
Yes	298	56.02	234	43.98					
No	2109	61.87	1300	38.13					
Betablocker					2.952	0.001	1	0.086	0.9802
Yes	992	62.71	590	37.29					
No	1415	59.98	944	40.02					
CCB					1.145	0.012	1	0.285	0.9138
Yes	565	59.60	383	40.40					
No	1842	61.54	1151	38.46					
Diuretic					0.169	0.004	1	0.681	0.9504
Yes	888	61.50	556	38.50					
No	1519	60.83	978	39.17					
PPI					0.301	0.005	1	0.583	0.9416
Yes	729	61.73	452	38.27					
No	1678	60.80	1082	39.20					
Statin					0.264	0.010	1	0.607	0.9211
Yes	925	61.58	577	38.42					
No	1482	60.76	957	39.24					

Note: Based on 3941 (adherent (N) = 2407 (61.08%)) older adults with dementia and newly diagnosed MDD. Abbreviations: PD: Parkinson’s disease; ELX: Elixhauser; ACE inhibitors: angiotensin-converting-enzyme (ACE) inhibitors; ARBs: angiotensin II receptor blockers; CCB: calcium-channel blockers; PPI: proton pump inhibitors. * Represents statistical significance (*p*-value < 0.05).

**Table 3:** Survival estimates (95% CI) for all-cause mortality and cumulative incidence estimates (95% CI) at 90, 180, 270, 365, 455, and 545 days for hospitalization and falls and fractures.

Acute Depression Treatment Phase				
All-cause mortality				
	Adherent		Non-adherent	
90 days	0.9463	(0.9377–0.9537)	0.9164	(0.8990–0.9310)
180 days	0.8891	(0.8775–0.8997)	0.8607	(0.8395–0.8794)
270 days	0.8367	(0.8232–0.8493)	0.8134	(0.7898–0.8346)
365 days	0.7857	(0.7708–0.7999)	0.7617	(0.7362–0.7851)
455 days	0.7418	(0.7259–0.7570)	0.7240	(0.6974–0.7488)
545 days	0.7050	(0.6883–0.7209)	0.6785	(0.6507–0.7046)
**All-cause hospitalization**				
	Adherent		Non-adherent	
90 days	0.1702	(0.1571–0.1840)	0.1998	(0.1762–0.2232)
180 days	0.2736	(0.2573–0.2893)	0.3209	(0.2933–0.3486)
270 days	0.3603	(0.3430–0.3775)	0.3910	(0.3626–0.4191)
365 days	0.4190	(0.4013–0.4362)	0.4667	(0.4370–0.4980)
455 days	0.4643	(0.4470–0.4813)	0.5077	(0.4765–0.5362)
545 days	0.5042	(0.4869–0.5216)	0.5438	(0.5127–0.5731)
**Falls and Fractures**				
	Adherent		Non-adherent	
90 days	0.0953	(0.0849–0.1058)	0.1027	(0.0852–0.1203)
180 days	0.1491	(0.1366–0.1616)	0.1500	(0.1290–0.1699)
270 days	0.1911	(0.1766–0.2044)	0.1897	(0.1687–0.2109)
365 days	0.2351	(0.2192–0.2493)	0.2412	(0.2167–0.2650)
455 days	0.2611	(0.2453–0.2771)	0.2689	(0.2425–0.2944)
545 days	0.2883	(0.2727–0.3044)	0.3008	(0.2731–0.3266)
**Continuation Depression Treatment Phase**				
**All-cause mortality**				
	Adherent		Non-adherent	
90 days	0.9430	(0.9328–0.9517)	0.9398	(0.9263–0.9509)
180 days	0.8887	(0.8753–0.9008)	0.8899	(0.8726–0.9049)
270 days	0.8344	(0.8187–0.8490)	0.8343	(0.8141–0.8525)
365 days	0.7895	(0.7723–0.8055)	0.7888	(0.7668–0.8090)
455 days	0.7473	(0.7289–0.7647)	0.7447	(0.7210–0.7666)
545 days	0.7014	(0.6811–0.7207)	0.7161	(0.6912–0.7394)
**All-cause hospitalization**				
	Adherent		Non-adherent	
90 days	0.1574	(0.1427–0.1728)	0.1739	(0.1565–0.1929)
180 days	0.2569	(0.2382–0.2752)	0.2796	(0.2579–0.3031)
270 days	0.3354	(0.3159–0.3552)	0.3619	(0.3385–0.3876)
365 days	0.3914	(0.3723–0.4119)	0.4243	(0.4011–0.4510)
455 days	0.4391	(0.4185–0.4601)	0.4674	(0.4437–0.4951)
545 days	0.4847	(0.4632–0.5056)	0.5012	(0.4772–0.5293)
**Falls and Fractures**				
	Adherent		Non-adherent	
90 days	0.0826	(0.0718–0.0937)	0.0778	(0.0651–0.0936)
180 days	0.1348	(0.1214–0.1491)	0.1338	(0.1162–0.1526)
270 days	0.1872	(0.1722–0.2043)	0.1815	(0.1620–0.2009)
365 days	0.2231	(0.2068–0.2407)	0.2187	(0.1981–0.2396)
455 days	0.2543	(0.2368–0.2725)	0.2555	(0.2336–0.2786)
545 days	0.2794	(0.2606–0.2979)	0.2829	(0.2600–0.3074)

Note: A point estimate and/or upper limit to the confidence interval will not be available when all the remaining individuals got censored.

**Table 4:** Median and first quartile survival times (95% CI) in days for all-cause mortality and time-to-hospitalization and falls and fractures (95% CI) in days.

Acute Depression Treatment Phase				
Mortality				
	Adherent		Non-adherent	
1st Quartile	441	(406–474)	385	(342–455)
Median	NA	NA	NA	NA
**Hospitalization**				
	Adherent		Non-adherent	
1st Quartile	153	(140–171)	122	(103–139)
Median	530	(499–587)	425	(364–492)
**Falls and Fractures**				
	Adherent		Non-adherent	
1st Quartile	424	(364–471)	403	(336–459)
Median	NA	NA	NA	NA
**Continuation Depression Treatment Phase**				
**Mortality**				
	Adherent		Non-adherent	
1st Quartile	445	(415–483)	443	(397–492)
Median	NA	NA	NA	NA
**Hospitalization**				
	Adherent		Non-adherent	
1st Quartile	169	(146–191)	152	(135–170)
Median	578	(536–666)	543	(463–599)
**Falls and Fractures**				
	Adherent		Non-adherent	
1st Quartile	436	(387–510)	440	(384–516)
Median	NA	NA	NA	NA

Note: A point estimate and/or upper limit to the confidence interval will not be available where an insufficient number of events have occurred.

## Supplementary Materials

The following are available online at https://www.mdpi.com/2077-0383/9/10/3358/s1, Figure S1: Propensity score distribution for adherent and non-adherent group during acute phase. Figure S2: Distribution of adjusted weights for adherent and non-adherent group during acute phase. Figure S3: Propensity score distribution for adherent and non-adherent group during continuation phase. Figure S4: Distribution of adjusted weights for adherent and non-adherent group during continuation phase.
