# Supplementary material for: Health Outcomes Associated with Adherence to Antidepressant Use during Acute and Continuation Phases of Depression Treatment among Older Adults with Dementia and Major Depressive Disorder
Source: J Clin Med. 2020 Oct 20;9(10):3358. doi: 10.3390/jcm9103358 (PMC7589937; doi:10.3390/jcm9103358)
Supplement: Supplementary file 1 [file jcm-09-03358-s001.pdf]

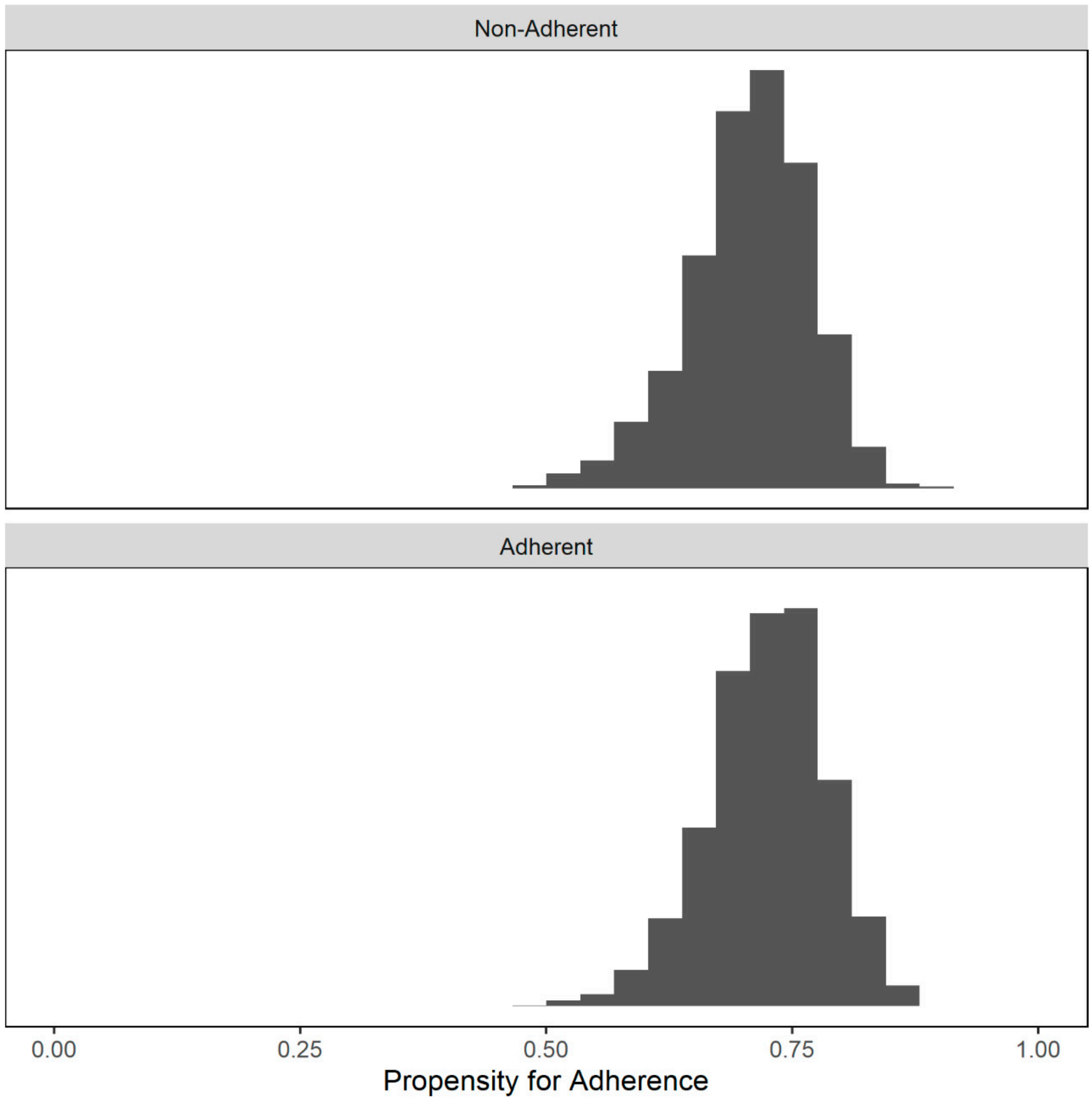

Figure S1. Propensity score distribution for adherent and non-adherent group during acute phase.

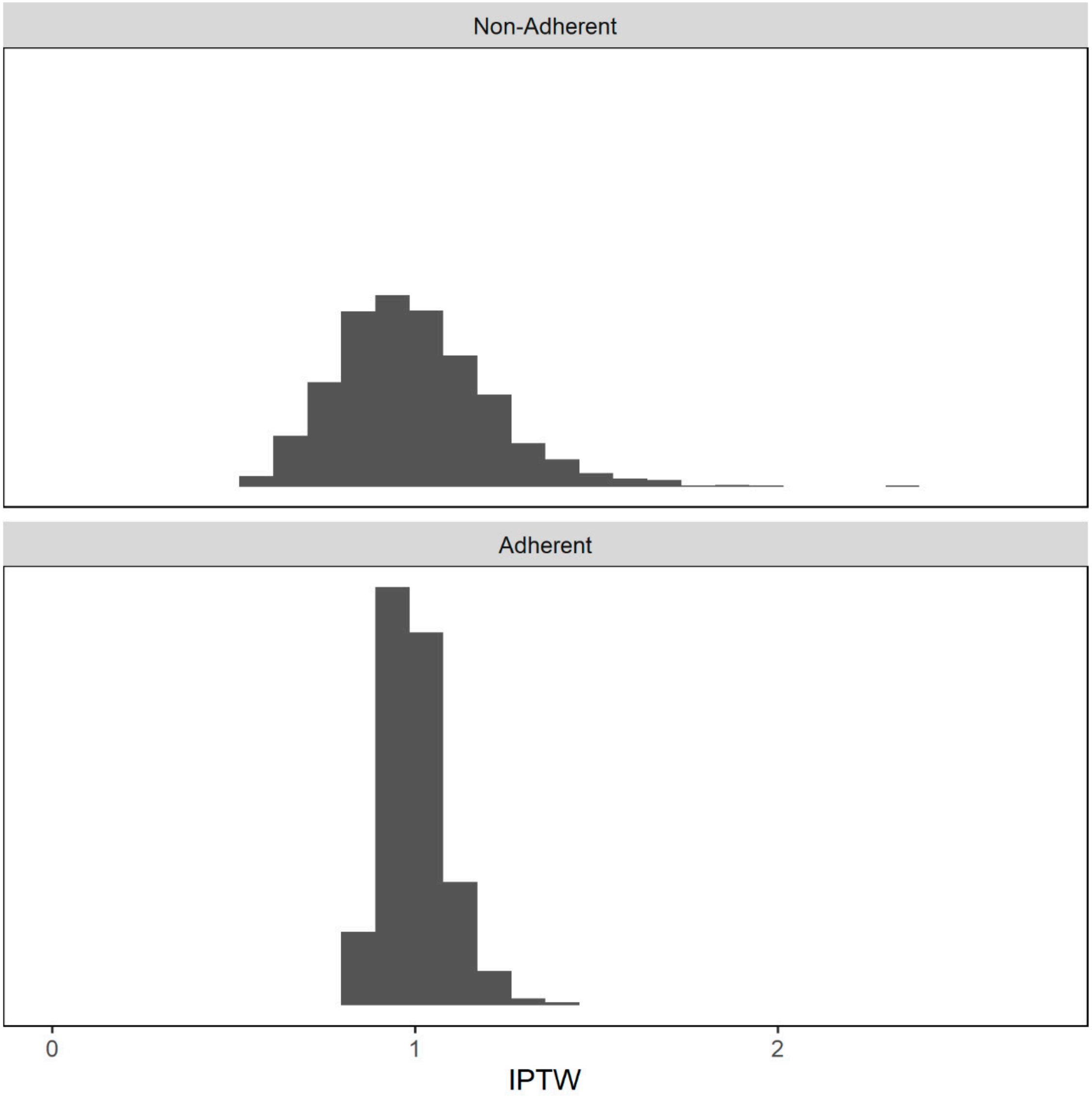

Figure S2. Distribution of adjusted weights for adherent and non-adherent group during acute phase.

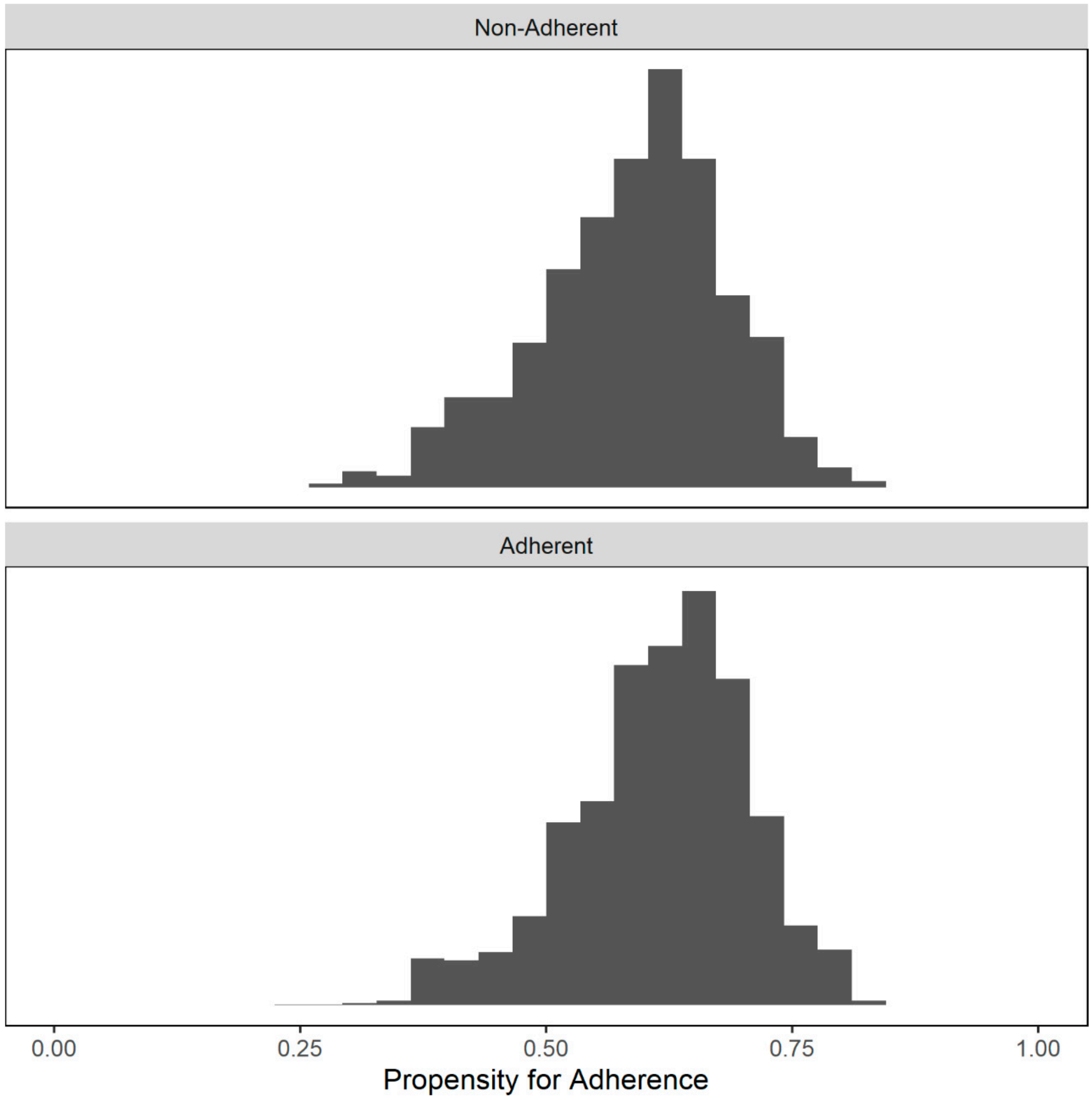

Figure S3. Propensity score distribution for adherent and non-adherent group during continuation phase.

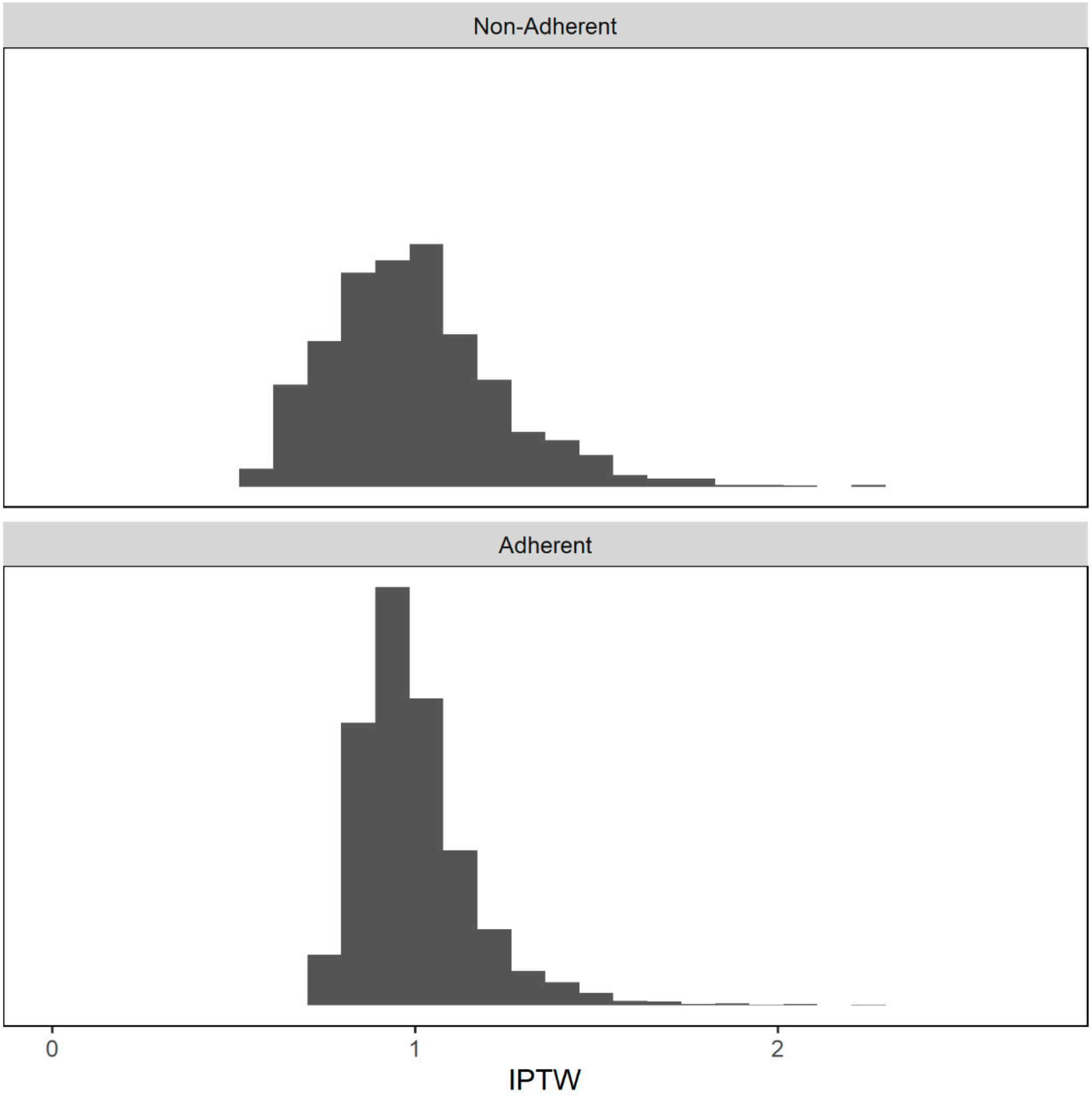

Figure S4. Distribution of adjusted weights for adherent and non-adherent group during continuation phase.
